# Societal costs of air pollution-related health hazards: A review of methods and results

**DOI:** 10.1186/1478-7547-6-19

**Published:** 2008-09-11

**Authors:** Tanjima Pervin, Ulf-G Gerdtham, Carl Hampus Lyttkens

**Affiliations:** 1 Health Economics Program (HEP), Department of Clinical Sciences, Malmö, Lund University SE-205 02 Malmö, Sweden; 2 Department of Economics, Lund University, SE-220 07 Lund, Sweden

## Abstract

This paper aims to provide a critical and systematic review of the societal costs of air pollution-related ill health (CAP), to explore methodological issues that may be important when assessing or comparing CAP across countries and to suggest ways in which future CAP studies can be made more useful for policy analysis. The methodology includes a systematic search based on the major electronic databases and the websites of a number of major international organizations. Studies are categorized by origin – OECD countries or non-OECD countries – and by publication status. Seventeen studies are included, eight from OECD countries and nine from non-OECD countries. A number of studies based on the ExternE methodology and the USA studies conducted by the Institute of Transportation are also summarized and discussed separately. The present review shows that considerable societal costs are attributable to air pollution-related health hazards. Nevertheless, given the variations in the methodologies used to calculate the estimated costs (e.g. cost estimation methods and cost components included), and inter-country differences in demographic composition and health care systems, it is difficult to compare CAP estimates across studies and countries. To increase awareness concerning the air pollution-related burden of disease, and to build links to health policy analyses, future research efforts should be directed towards theoretically sound and comprehensive CAP estimates with use of rich data. In particular, a more explicit approach should be followed to deal with uncertainties in the estimations. Along with monetary estimates, future research should also report all physical impacts and source-specific cost estimates, and should attempt to estimate 'avoidable cost' using alternative counterfactual scenarios.

## Introduction

Air pollution is one of the most serious environmental problems in urban areas around the world [1]. The rapid process of urbanization and extensive energy utilization (mostly due to rapid economic expansion and population growth over the past few decades) has made urban air pollution a growing problem [2]. The air contains varying levels of pollutants originating from motor vehicles, industry, housing, and commercial sources. The effects of air pollution have multifaceted consequences for human welfare in areas such as health, agriculture, and the ecosystem. Notably, numerous studies have shown that air pollution adversely affects human health. It is well known that criteria air pollutants (criteria pollutants are the non-toxic air pollutants which are considered most responsible for urban air pollution and are known to be hazardous to health), namely carbon monoxide (CO), nitrogen dioxide (NO_2_), particulates (the concentration of particles of various sizes in the air can be measured as micrograms per cubic meter- μg/m^3^. PM_10 _and PM_2.5 _are expressed particles of sizes 10 μg and 2.5 μg or less, i.e., PM_10 _and PM_2.5_), sulfur dioxide (SO_2_), and ozone have serious impacts on health [3]. Epidemiological evidence supports an association between exposure to these ambient air pollutants and various health effects, such as respiratory symptoms or illness (e.g. asthma), impaired cardiopulmonary function, reduction of lung function, and premature mortality [4,5]. In particular, the most serious health impacts include a significant reduction in life expectancy, and premature death, both of which are strongly linked to exposure to PM [6]. Although exposure to air pollution damages the health of everyone, numerous studies have shown that certain groups of vulnerable people (e.g. elderly people, children, and those with underlying disease) are at greater risk of being affected by air pollutants. Additionally, many recent health studies increasingly support the hypothesis that poor indoor environment, tobacco smoke, and combustion emissions not only cause respiratory and cardiovascular diseases, but may also cause premature death [7].

In health economics, it is rather common to use the cost-of-illness (COI) framework to quantify the costs of different health risk factors (e.g. air/noise pollution, smoking, drug/alcohol addiction etc) in monetary terms. COI studies are not full economic evaluations because they do not include comparisons of alternative interventions/programs [8]. Instead, COI studies estimate the burden of diseases and other adverse conditions or events on society or parts of society.

Cost is the value of a resource, defined as the value that could be gained by using the resource in an alternative way. In the societal perspective, a COI study includes all costs, no matter who incurs them. For example, transfers such as taxes, social allowances, and insurance premiums are not considered a societal cost as they do not affect the amount of resources available in the society [9]. However, there is still a societal cost connected to the transfer payments (i.e., the administrative cost that is an actual resource consumption), the deadweight loss due to a tax is different from the administrative cost of the tax system; it is the loss of welfare due to the tax distorting prices and consumption. The deadweight loss will be different with different tax schemes.

The costs are estimated in four steps: *firstly*, the relevant resources are identified; *secondly*, these resources are quantified (e.g. days in hospital, visits to the doctor, etc.); *thirdly*, the quantified resources are monetized at their opportunity cost; and *finally*, costs not occurring in the same period of time are discounted.

Costs in COI are mainly divided into three broad categories: direct costs, indirect costs, and intangible costs. Direct costs include both direct health care costs (e.g. the costs of medicines, diagnostic tests, supplies, health care personnel, and hospital facilities) and direct non-health care costs (e.g. the cost of caregivers' time, injure crops and forest, material- damage cost, and visibility cost; informal care is an important component of direct non-health care costs). Productivity costs are often termed "indirect costs". This cost component includes: (i) costs associated with loss of productivity or impaired ability to work due to morbidity and (ii) loss of productivity due to death. The intangible costs are non-marketable resources which reflect the patient's level of pain and suffering, and the limitations imposed by this pain and suffering on the patient's quality of life. COI studies can be designed either as top-down studies or as bottom-up studies, depending on the data material. A top-down study estimates costs for a given population sample using statistical databases and/or registers, whereas bottom-up studies measure costs from a patient sample and extrapolate this to the population. Both approaches have their own problems; the former because not all costs for a certain disease/condition can usually be found in registers, and the latter because the patient sample needs to be unbiased and representative of the whole population [10].

A COI study can be either prevalence-based or incidence-based. Given a specific population, a prevalence-based study estimates present and future costs resulting from diseases/conditions or treatments that occurring during a given period of time. Incidence-based studies, on the other hand, measure the lifetime cost of diseases/conditions. Incidence-based studies are more appropriate when measuring the effect of particular interventions, while prevalence-based studies are useful for planning and budget decisions. The main shortcoming of incidence-based studies is that they require considerable knowledge and information about the disease/condition in question and the costs that occur as a result thereof. This is a major problem, especially in a COI study dealing with societal phenomena, and so a prevalence-based study is often the better choice in some practice [10]. However, COI studies may use a mixture of prevalence-based and incidence-based approaches, with some costs attributed to the year under study and other costs occurring in the future.

COI studies are not beyond criticism. One line of criticism is that COI studies are founded on a weak theoretical basis and cannot be used in the prioritization of resources, thus limiting their use as a health policy tool [11]. For example, COI studies fail to evaluate the effectiveness of particular policies or programs, and give no help in deciding how to divide resources efficiently between alternative interventions [12,13]. Moreover, the use of different data and methods in different studies means that it may be difficult to compare findings across studies. Another line of criticism of the COI framework is that it generally presents conservative estimates because it often excludes certain cost dimensions associated with different risk factors (e.g. research costs, costs of prevention programs, costs of introducing new technology, maintenance costs, and so on) [14].

Nevertheless, traditional COI studies are still valuable, since they can identify any large gaps in the knowledge and data which would be required for a full accounting of costs, and they may stimulate new data collections and analyses aimed at filling these gaps. By identifying the different components of cost, and estimating their size, COI studies may provide some ideas of the order of magnitude of the social and health problems resulting from the disease or condition in a particular society or locality, particularly if studies with comparable methods have been carried out elsewhere or on other diseases or conditions in the same society or locality. Moreover, COI studies can provide policy makers with potentially useful information for use in determining research and funding priorities for how healthcare money should be spent during a certain period, as well as assisting in budget planning decisions [15,16]. Finally, by providing source-specific cost estimates for a particular risk factor (e.g. the costs associated with vehicle-induced air pollution), COI studies also pave the way for cost-effectiveness analysis by identifying the main causes within a risk factor (e.g. the extent of vehicle-induced air pollution), and can become useful sources of policy-relevant information.

The aim of this paper is three-fold. Firstly, we systematically review the evidence regarding the societal costs associated with air pollution (CAP). Secondly, we explore methodological issues that may be important when assessing or comparing CAP across countries. Thirdly, we suggest ways in which future CAP studies can be made more useful for policy analysis.

## Methods of the review

### Search strategy and inclusion criteria

Systematic searches in electronic databases were carried out for articles published between 1980 and the end of June, 2006. MEDLINE (via PubMed), EconLit, and the International Bibliography of the Social Sciences (IBSS) (via CSA) were used for published papers. The websites of international institutions, namely the World Bank and World Health Organization, were used as additional sources of literature. Since only a limited number of studies have been published in the field of CAP, manual searches for unpublished literature were also performed on a number of other sites, for example, the European Commission's Externalities of Energy (ExternE) project, the United States Environmental Protection Agency (USEPA), The Institute of Transportation Studies (located at the University of California, Davis, the institute produces considerable studies of the societal cost of motor vehicles that remains the most comprehensive works ever done based on the USA data) and the Ontario Medical Association (OMA). The search used the following key words: "air pollution" AND "social costs" OR "welfare costs" OR "external costs" OR "cost of illness" OR "economic costs". Studies published in languages other than English were excluded from the review, as were those that did not use quantitative methodology, those that did not estimate health damage in monetary terms, those that used methods other than a traditional COI or willingness-to-pay (WTP) framework, and those that estimated only the short-term effects of CAP (e.g. time series-based studies, which might be unable to capture the costs of reduced life expectancy due to long-term morbidity; see e.g. [17]).

### Analytical strategies

The studies were divided into two groups according to whether the data came from OECD or non-OECD countries. They were also divided according to publication status, that is, whether or not they were published in peer-reviewed journals. It should be noted that some of the unpublished studies that based on the Externalities of Energy project, the Green Accounting Project I & II (e.g. ExternE, GARP I, and GARP II studies and reports by the Institute of Transportation Studies, University of California, Davis) had not been presented in the tables (as they followed identical methods), but instead summarized them separately in the text.

The analysis was split into three main parts. Firstly, the characteristics of each study were described: study perspective, type of analysis, data sources, sample size, and approach (i.e. top-down or bottom-up). These characteristics are summarized in Table 1. Secondly, since the estimation of productivity losses and intangible costs (often not included in typical COI studies) may be critical, special focus was given to the methods used to estimate these in the different studies. Each study was examined to identify the methodological characteristics that were followed in estimating CAP; these methodological aspects are summarized in Table 2. Finally, Table 3 summarizes in detail the estimated total societal costs and components. All costs were converted into a common currency, the US dollar (using nominal exchange rates). If a study did not report per capita cost, we estimated it based on the available information.

**Table 1:** Summary of study characteristics.

**Study**	**Country**	**Study Year**	**Data Source(s)**	**No. of Observations**	**Perspective**	**Incidence/Prevalence**	**Top-down/Bottom-up**	**Sensitivity analysis**
**Published Studies: OECD Countries**								

Zmirou *et al*. [18]	France	1994	Primary data: A cross-sectional study conducted in three cities in France.	970,000	Societal	Prevalence	Bottom-up	Yes (low and high)
Voorhees *et al*. [19]	Tokyo, Japan	1994	Secondary sources: Tokyo Metropolitan Government (TMG), Japanese Environment Agency (JEA), Japanese Ministry of Transportation.	Not stated	Societal	Prevalence	Top-down	Yes
Navrud [22]	Norway	1996	Primary data: A CV survey conducted in Norway & also use other secondary data sources	1009	Societal	Prevalence	Combination of bottom-up & top-down	Yes
Rozan [20]	Strasbou, France	1998	Primary data: A survey conducted in Strasbourg in France. Some epidemiological studies are also used as a secondary source.	1,000	Societal	Prevalence	Bottom-up	No
Neidell [21]	California, USA	1998	Secondary sources: California Hospital Discharge Data (CHDD), US Environmental Protection Agency (EPA), National Climatic Data Center, Census of Population, 1990, Air Resources Board, 1990	800,000 (Children aged 1–18)	Societal	Prevalence	Top-down	Yes (low and high)
Panis [23]	Belgium	1998	Data sources: Used different secondary sources, e.g., ExternE project data are used.	Total population of Belgium	Societal	Prevalence	Top-down	No

**Unpublished Studies: OECD Countries**								

DSS Management Consulting inc.) [24]	Canada	2000–2015	Data sources: Statistics of Canada & Census Information, hospital-level survey conducted by the Ontario Medical Association (OMA).	11 million (total population of Ontario)	Societal	Prevalence	Combination of bottom-up & top-down	No
Vergana and the Mexico Air Quality the WB study [25]	Metro-politan Mexico City (ZMV)	1999	Secondary sources: Mexican National Institute of Statistics, Geography & Information (INEGI), National Health Survey, 1994	17 million	Societal	Prevalence	Top-down	Yes (high, central and low)

**Published Studies: non-OECD Countries**								

Larson *et al *[45]	Volgograd Russia	1995	Secondary data: 29 stationary sources	Total population 50,000* 29 = 1,450,000	Societal	Prevalence	Top-down	Yes
Alberini & Krupnick [1]	Taiwan	1991–1992	Primary data: A combined epidemiological & economic study conducted in three cities in Taiwan.	Total population: 3,031,532 Sample observations: 87,676	Societal	Prevalence	Bottom-up	No
Srivastava & Kumar [2]	Mumbai, India	1997	Sources: Institute for Population Sciences, Mumbai, Transport Commissioners office, Maharashtra State, Mumbai.	15.6 million	Societal	Prevalence	Top-down	No
Quah & Boon [50]	Singapore	1999	Secondary data sources: ENV Annual Report, 1998, Monthly Digest of Statistics, 1999, Singapore Dept. of Statistics, Ministry of Health, Singapore.	Total population in Singapore = 3,893,600	Societal	Prevalence	Top-down	Yes (high, central & low)
Resosudarmo& Napitupulu [48]	Indonesia, Jakarta	1998	Data sources: Indonesian Central Statistics Body (BPS), a survey conducted at Cipto Hospital (public hospital), and another survey conducted at Universitas Kristen Indonesia Hospital (private hospital) and at several individual medical practices.	Total population in Jakarta = 11 million	Societal	Prevalence	Combination of bottom-up & top-down	No
Kan & Chen [46]	Shanghai, China	2001	Data sources: Shanghai Municipal Environmental Protection Bureau, Shanghai Environmental Monitoring Center, Shanghai Municipal Bureau of Public Health, China Ministry of Health.	Total urban population of Shanghai	Societal	Prevalence	Top-down	No
Deng [47]	Beijing, China	2000	Data sources: Primary data Secondary sources: WHO, World bank, National Bureau of Statistics of China, Beijing Environment Protection Bureau, China Statistical Yearbook	Total population of Beijing = 13.82 million	Societal	Prevalence	Combination of bottom-up & top-down	Yes

**Unpublished Studies: non-OECD Countries**								

Saksena & Dayal [49]	India	1997	Secondary Sources: Central Pollution Control Board (CPCB), Central Bureau of Health Intelligence (CBHI).	Total population in India = 846 million (used 1991 census)	Societal	Prevalence	Top-down	Yes (Low & High)
Report of Environment Protection Department, Hong Kong [51]	Hong Kong, China	1997–1998	Sources: Report on Focus Group Survey Data, Hospital Authority (HA), Department of Health, Census & Statistics Department, and Government Gazette.	Total population in Hong Kong = 6.31 million (estimated in 1996)	Societal	Prevalence	Combination of bottom-up & top-down	Yes (ranging numerical)

**Table 2:** Summary of studies emphasizing methodological characteristics.

**Study**	**Components of Air Pollution**	**Mortality & Morbidity ** (Types of Diseases)	**Cost Components and Estimation method**	**Approach(s) used for estimating productivity Losses**	**Discount rate**
**Published Studies: OECD Countries**					

Zmirou *et al *[18]	PM_10_	Morbidity: Asthma & other respiratory conditions or symptoms.	Direct medical costs: Drug consumption, medical and other health professionals' care, biological or radiological examinations, daily hospital costs. Indirect costs: Work absence due to illness (adult males), work absence for child care (mothers), days of school absence. (Wage losses have converted into average daily wage losses.) Method: A figure of 970,000 inhabitants (three cities in France) is multiplied by the average unit cost of asthma & other respiratory conditions.	Production loss (due to morbidity) is valued using HCA. VOSL is used to evaluate premature mortality cost.	Not stated
Voorhees *et al *[19]	Nitrogen dioxide (NO_2_)	Morbidity: Phlegm & sputum in adults, lower respiratory illness in children.	Direct costs: Direct medical costs. Indirect costs: Costs of lost workers' wages, costs due to mothers' wage losses due to caring for sick children. Method: Average cost is multiplied by population.	Production loss is valued using work days lost (including mothers' workdays lost due to looking after sick children) multiplied by wage.	Not stated
Panis [23]	SO_2_, NO_x _& PM	Morbidity: Respiratory minor illness, serious respiratory & cardiovascular illness	Not stated separately Method: Adopted Impact path way approach from ExtrenE Project	N/A	Not stated
Navrud [22]	PM_10_, PM _2.5_, NO_x_, O_3_	Morbidity: Seven light symptoms	Direct Costs: medication, Doctor's & hospital visits Indirect costs: cost of wage earning lost due to RADs & mortality Intangible cost: restricted leisure activities Method: Intangible costs are estimated using CVM	N/A	Not stated
Rozan, [20]	Air pollution (the specific pollutant was not identified)	Morbidity: Minor illness only, hospitalization not relevant.	Direct costs: Medical treatment. Indirect costs: Wage loss due to sick leave. Intangible costs: Pain, suffering, loss of opportunity to practice leisure activities due to illness. Method: Intangible costs are estimated using CVM.	Production loss is valued using HCA.	Not stated
Neidell [21]	Carbon monoxide (CO)	Morbidity: Asthma (children).	Direct costs: Hospitalization costs. Method: Average charges for ER admission for asthma are multiplied by the number of admissions.	N/A	Not stated

**Unpublished Studies: OECD Countries**					

DSS Management Consulting inc. [24]	Ozone & PM_10_	Morbidity: Respiratory & cardiovascular illness.	Direct costs: Hospital admission, emergency room visits, doctor's room visits, medication, mortality. Indirect costs: Lost productivity. Intangible costs: Value of pain & suffering. Method: To estimate the total cost, the total population of Ontario is multiplied by the average cost.	HCA.	Not stated
Vergana and the Mexico Air Quality the WB study [25]	PM_10 _& Ozone	Mortality. Morbidity: Respiratory diseases (cardiocerebrovascu-lar, congestive heart failure), Asthma. Chronic morbidity: Chronic bronchitis & chronic cough, prevalence (children).	Direct costs: Medication, hospital admission, emergency room visits. Indirect costs: Restricted activity days, work days lost (adults), work days lost by women due to RAD in children. Method: To estimate the total cost, the population of 17 million is multiplied by the average cost.	To estimate premature mortality cost, this study has followed ExternE(1999) approach. & assumed the number of premature deaths equal to Years of Life Lost (YOLL) about 0.75 years.	3%

**Published Studies: Non-OECD Countries**					

Larson *et al *[45]	PM_10_	Mortality risks.	Indirect costs of mortality. Method: Among Volgograd's population of 50,000 people, the annual number of deaths is estimated at 2666.88. Annual mortality costs were multiplied by the total population in order to obtain the total costs of mortality.	VOSL is estimated using HCA.	10%
Alberini & Krupnick [1]	PM_10_	Morbidity: 19 minor respiratory-related symptoms such as cold, sore throat, headache, eye irritation, etc.	Direct health care costs: Doctor's fees, prescription medication. Indirect health care costs: Earning loss due to absenteeism, restricted activity days. Method: To estimate the total COI, average unit cost associated with every cost components (i.e. doctor's visits Medication costs, and earning lost) are multiplied with total number of adult residents of three Taiwan's cities and has added them together.	Work days losses are estimated using HCA. WTP is used to evaluate premature mortality.	Not stated
Srivastava & Kumar [2]	NO_2_, CO, HC, PM (below 10 Micron)	Mortality and Morbidity: Chronic bronchitis, bronchitis in children, asthma, respiratory symptoms & illness.	Direct costs: Emergency room visits, hospital admission. Indirect costs: Loss of salary due to mortality & restricted activity days. Method: Average income loss due to morbidity & mortality is multiplied by the total population.	Production losses are estimated using HCA, WTP was used to estimate the monetary values of premature mortality, and cost is evaluated using VOSL.	5%
Quah & Boon [50]	PM_10_	Mortality. Morbidity: Asthma, respiratory symptoms, lower respiratory illness (LRI) in children, chronic bronchitis.	Direct costs for morbidity: Emergency doctor's room visits, Hospital admission. Indirect costs: Premature mortality & restricted activity days. Method: Unit costs are multiplied by population.	Production losses due to morbidities are estimated using HCA, WTP is used to estimate the monetary values of premature mortality, and cost is evaluated using VOSL.	3%
Resosudarmo & Napitupulu [48]	PM_10_, NO_2_, SO_2_	Premature mortality. Morbidity: Asthma attacks, chronic bronchitis, respiratory symptoms in children, chest discomfort in adults.	Direct costs: Hospital admission, emergency room visits. Indirect costs: Cost of premature death & restricted activity days. Method: Average cost per case is used to estimate the direct cost.	Both of HCA &VOSL are used (Mortality cost is evaluated using VOSL).	5%
Kan & hen [46]	PM_10_	Premature mortality. Morbidity: Asthma attacks (children and adults), chronic bronchitis, Acute bronchitis, respiratory illness, cardiovascular disease.	Direct costs: Hospital admission, outpatient visits, medication. Indirect costs: Cost of premature death & restricted activity days. Method: Both COI and WTP are used to estimate the direct & indirect costs.	WTP is used to estimate the monetary values of premature mortality and cost is evaluated using VOSL.	Not stated
Deng [47]	PM_10_	Mortality. Morbidity: Respiratory diseases, Cardiovascular, Lower respiratory infection/child asthma, Asthma (adult), Bronchitis, Chronic bronchitis Respiratory symptoms	Direct costs: Hospital admission, outpatient visits, emergency room visits. Indirect costs: Cost of premature death & restricted activity days. Method: Both COI and WTP are used to estimate the direct & indirect costs.	The study has adopted VOSL from WHO's estimation and then adjusted it by the ratio of Beijing's per capita GDP	Not stated

**Unpublished Studies: Non-OECD Countries**					

Saksena & Dayal [49]	PM_10_	Premature death. Morbidity: Respiratory symptoms, lower respiratory illness, asthma, chronic bronchitis	Direct costs: Hospital admission, emergency doctor's room visits. Indirect costs: Cost of premature death & restricted activity days. Method: Total population is multiplied by the unit values of health damage.	Both of HCA &VOSL are used (Mortality cost is evaluated using VOSL).	5%
Report of Environment protection Department, Hong Kong [57]	NO_2_, SO_2_, Rsp, & Ozone (O_3_)	Mortality & Morbidity: Respiratory diseases, cardiovascular diseases	Direct costs: Self medication & any other related expenses, Hospital admission, consultation fees (public and private), registration charges. Indirect costs: Wage loss due to illness & mortality. Method: Both COI and WTP are used to estimate the direct & indirect costs.	HCA. Mortality cost is evaluated using VOSL	7%

**Table 3:** Summary of the total societal cost by cost component.

**Study**	**Cost Components**				**Total Societal Cost**	**Per Capita CAP**	**CAP as % of GDP**
				
	**Direct Health care**	**Direct non-health care**	**Productivity Losses**	**Intangible Costs**			
**Published Studies: OECD Countries**							

Zmirou *et al *[18]	US $6.60–11.25 million (1 French franc = $0.17 in 1994)	Not estimated.	$5.10–8.72 million	Not estimated.	US $13.43–22.95 million (1 French franc = $0.17 in 1994)	US $13.85–23.66	Not stated.
Voorhees *et al *[19]	US $6,860 million	US $833 million	US $6,330 million	Not estimated.	US $14,023 million	N/A	Not stated.
Navrud [22]	Navrud estimated mean WTP per person for seven selected symptoms or illness and found that WTP per person was $376 for avoiding one additional day of symptoms, and about $1210 for 14 additional days per year.						
Rozan [20]	Doctor's visit: about US $24.91 per patient. Drug cost: US $74.75 per patient.	Not estimated.	US $21,299.00 average wage loss per adult patient (yearly). The yearly cost per student due to absenteeism is about US $3787.37.	Mean WTP is US $46.83 On average, 50% of total cost.	Information given in the paper does not permit calculation of the total cost.	N/A	N/A
Neidell [21]	US $5.2 million	Not estimated.	Not estimated.	Not estimated.	US $5.2 million	US $6.50	Not stated.
Panis [23]	Not Stated separately	Not Stated separately	Not Stated separately	Not Stated separately	$1.5 billion (approx.)	N/A	N/A

**Unpublished Studies: OECD Countries**							

DSS Management Consulting inc. [24]	US $674 million (approx) Can $1 = US $0.674	Not estimated.	US $2,696 million (approx)	US $3,370 million (approx)	US $6,740 million (approx)	US $612.73	Not stated.
Vergana and the Mexico Air Quality the WB study [25]	Total direct costs are not reported.	Not reported.	Total indirect costs are not reported.	Not reported separately.	US $760 million (approx) (in 1999 US dollars)	US $44.71	Not stated.

**Published Studies: Non-OECD Countries**							

Larson *et al *[45]	Not estimated.	Not estimated.	US $28.8–80.01 million (in 1997 US dollars)	Estimated but not mentioned figure.	US $28.8–80.01 million (in 1997 US dollars)	US $19.86–55.18	Not stated.
Alberini & Krupnick[1]	US $510,491 (PM_10 _= 100 μg/m^3^) to US $804,298 (PM_10 _= 350 μg/m^3^) (in 1992 US dollars)	Not estimated.	US $117,575–244,477 (in 1992 US dollars)	Estimated but not mentioned figure.	US $628,074–1,048,775 (in 1992 US dollars)	US $0.21–0.35	Not stated.
Srivastava & Kumar [2]	US $232.34 million (in 1997 US dollars) (1 Indian Rs = US $0.0275)	Not estimated.	US $76.32 million (1 Indian Rs = US $0.0275)	Not estimated.	US $308.66 million (1 Indian Rs = US $0.0275)	US $19.79	Not stated.
Quah & Boon [50]	US $1,889 million (for morbidity). (in 1992 US dollars)	Not estimated.	US $1,773 million (for mortality)	Estimated but not mentioned figure.	US $3662 million (in 1992 US dollars)	US $940	4.31%
Resosudarmo & Napitupulu [48]	US $115 million (approx) (in 1998 US dollars)	Not estimated.	US $65 million (approx)	Not estimated.	US $180 million	US $16.36	1%
Kan & Chen [46]	US $67.82 million	Not estimated	US $557.58 million	Estimated but not mentioned figure.	US $625.4 million	US $98.96	1.03%
Deng [47]	US $776.576(WTP) & US $180.265(HCA)	Not estimated	US $29.516 million (HCA)&US $197.101 million (WTP)	Estimated but not mentioned figure.	$974 million (according to WTP) & $210 million (according to COI)	$70.48 (according to WTP) & $15.20 (according to COI)	3.26%

**Unpublished Studies: Non-OECD Countries**							

Saksena & Dayal [49]	US $199.11 million (in 1995 US dollars) (1 Indian Rs = US $0.0253)	Not estimated.	US $18,783.99 million (in 1995 US dollars)	Not estimated.	US $18,983.10 million (in 1995 US dollars)	Lower estimates: US $2,000 for females and US $1,400 for males	Not stated.
Report of Environment protection Department, Hong Kong [51]	US $33.02–57.79 million (in 1998 US dollars) (1 HK $ = US $0.129)	Not estimated.	US $437.66–462.43 million (in 1998 US dollars) (1 HK $ = US $0.129)	Estimated but not mentioned figure.	US $495.45 million (approx) (in 1998 US dollars) (1 HK $ = US $0.129)	US $78.52	0.35%

## Results

### Search results

In total, 269 hits were produced from the selected databases. From the initial searches, articles were excluded where the title and abstract made it clear that the paper did not fulfill the inclusion criteria. After exclusions, 31 relevant articles/reports were initially identified as potentially fitting the selection criteria; 11 from EconLit and IBSS, 16 from PubMed, one from the WB and three reports (one published article) from the Institute of Transportation Studies (University of California, Davis). Since some of these studies appeared in more than one database, finally we ended up with 17 articles from 14 different countries (excluding ExternE studies, GARP I & GARP II, and the articles and reports produced by the Institute of Transportation Studies); all these 17 studies are summarized in the tables. As shown in Tables 1, 2, 3, 13 of the 17 studies were published and 4 were unpublished. Eight of the studies (six published, two unpublished) used data from OECD countries, and nine (seven published, two unpublished) used data from non-OECD countries.

### Studies based on the OECD countries

All eight OECD studies [18-25] had a societal perspective, all were prevalence-based studies (see Table 1), and except one unpublished study [25], all focused on the morbidity impacts of air pollution. However, different studies looked at different pollutants and different diseases. While all eight studies estimated the direct health care costs, they all ignored different cost components within the category of direct costs, except for Zmirou *et al*. [18]. None of these studies calculated all the direct non-health care costs (e.g. travel costs, time costs, and the costs of special diets), even though these costs seem to be an important part of total CAP. All authors, except for Neidell [21], estimated the indirect cost of working days lost using the human capital approach (HCA), the value of statistical life (VOSL) (VOSL is estimated as the discounted value of expected future income at the average age. The value of a statistical life should not be confused with the value of a human life) approach, or both. Moreover, Zmirou *et al*. [18], Voorhees *et al*. [19], and Vergana *et al*. [25] also estimated one of the major components of non-direct health care costs, namely the cost of mothers' earnings lost due to caring for sick children, a cost component that is often overlooked in traditional COI studies (see Table 2); however, only Voorhees *et al*. [19] reported this cost component separately. Only three studies; those by Rozan [20] and Navrud [22], and one unpublished study (Ontario Medical Association Study [24]), attempted to estimate the intangible cost component- a cost component generally ignored in COI studies. These studies used a willingness-to-pay (WTP) approach to assess the intangible costs (cost of disutility due to pain, suffering, and the loss of opportunities to practice leisure activities, etc.). For example, Rozan [20] used econometric techniques to predict that the mean WTP, which was considered as the estimation of the intangible costs, would on average make up 50% of the total costs. In the OMA study [24], which also used the WTP approach to estimate the costs of pain and suffering, these intangible costs were again found to make up about 50% (5 billion) of total CAP. Navrud [22] followed a contingent valuation approach similar to that of Tolley *et al*. [26], but using an improved version of the survey and sample design; he found that there was a declining marginal value of a symptom or illness day per year: per person the mean WTP of $376 to avoid one additional day of symptoms, while about $1210 to avoid 14 additional days symptoms. Navrud [22] also attempted to compare his study results with other European studies, running across a number of problems in the process. For example, without specifying the number of avoided days of symptoms or illness, Rozan [20] found the mean WTP of about $46.83 to avoid minor illness or symptoms per household, per year in France. It was difficult to compare the findings that reported by Navrud with Rozan because the number of avoided days was not specified by Rozan.

As expected, due to wide variations in methods (e.g. different pollutants and exposure levels, different functions and cost estimation methods), there are huge variations in estimated costs, both across OECD countries and between different studies within a country (see Table 3). For example, Zmirou *et al*. [18] estimated per capita CAP as ranging between $13.85 and $23.66 in three metropolitan areas of the Rhône-Alpes region in France, whereas Rozan [20] estimated the mean WTP for avoiding disutility due to morbidity (a component of total societal cost) at half of the total cost, or about $46.83, in Strasbourg, France.

### Studies based on the ExternE methodology

The ExternE project is concerned with the estimation of the marginal external cost of air pollution caused by vehicles for different areas in Europe [e.g. [27]]. To calculate the external costs of airborne pollutants, they use the "Impact Pathway" methodology, in which dispersion models and dose-response functions (DRFs) are employed to estimate health impacts. Notice that, DRFs are used to look at the statistical relationship between air pollution and human health outcomes. Most of the epidemiological studies linking air pollution and health endpoints are based on a relative risk model in the form of Poisson regression. Due to lack availability of epidemiological studies based on a country's own data, the majority of the studies around the world have had to rely on few international studies those have been conducted in the USA and Europe (e.g. the American Cancer Society study [28], or the Six U.S. Cities Study [29]). The monetary valuation of the health effects is conducted by a WTP method, and the costs associated with mortality are estimated using the VOSL approach. In an unpublished report based on the ExternE methodology, Nocker *et al*. [30] assessed the life cycle impacts on human health and the environment (i.e. agriculture, material, and the ecosystem, but without monetizing the ecological impact) for Belgium in 1998–2000. Different pollutants were examined (e.g. SO_2_, NO_x _through nitrates and ozone, and PM) and the total costs of mortality and morbidity were estimated to be approximately between $2.56 and $2.92 billion. Nocker *et al*.[30] also reported that mortality and morbidity costs were dominant in total CAP, 98% of costs came from mortality and morbidity, with the other 2% coming from agriculture and material damage due to SO_2_, and these costs were mainly caused by petrol and diesel cars.

Another pair of research projects funded by the European Commission, known as *Green Accounting Research Project I & II *(GARP I & GARP II), also drew heavily on the ExternE methodology. The projects estimated the impact of air pollution in four different European countries: Germany, Italy, UK, and the Netherlands [see [31,32]]. Two main elements were considered in the analysis: damage calculation and damage attribution. Damage calculation was performed using a computer model known as the ECOSENSE model; it involved combining pollutant concentration and population maps in order to calculate the value of the damage caused by the pollution on human health, crops, and building materials. The main pollutants considered were PM_10_, SO_2_, and ozone, and the estimation was based on the WTP approach. Three major health impacts were estimated, namely chronic mortality, chronic bronchitis, and restricted activity days caused by PM_10_. The results show that pollution-related damage cost about 2.8% of GDP for Germany, 4.4% for Italy, 3.9% for the Netherlands, and 2.0% for the UK in 1994. Health damage represented by far the largest share of damage costs in each country. Project GARP I estimated that the damage costs in 1990 comprised 4.1% of GDP for Italy, 5% for the Netherlands, and 3.3% for the UK. However, the authors point out that these values are not directly comparable over time, because the exposure-response functions and valuation methods differed between the two time points. For example, in the earlier phases of ExternE projects [see [33,34]], VOSL was valued at around €3 million; however, a later contingent valuation study carried out in Europe led to this value being lowered to €1 million [35].

### Studies based on the USA data

Based on the USA data, Keeler and Small's [36] study is one of the most influential and widely cited works on the costs of automobile use. In particular, it is one of the first attempts to quantify the non-market costs of automobile use, such as time cost, maintenance cost. However, most of the costs reported in this study are now outdated, and many of the methods have been improved [37].

Based on the USA studies, an outstanding review of the literature on the societal cost of motor-vehicle use can be found in Murphy and Delucchi [37]. In doing the review, the authors highlighted the study's aim, scope, conclusions, and summarized the cost estimates by different individual cost categories. The studies included in their review were also assessed by the degree of originality and the extent of the detail of each major cost estimates. Based on their review the authors concluded that many of the estimates included in the studies were based on literature review rather than detailed analysis. Other problems they identified that many of the studies were outdated, superficial, non-generable or otherwise inappropriate.

The Institute of Transportation Studies based at University of California, Davis produced several detailed studies on the social costs of motor-vehicle related air pollution, which seemed to be the most comprehensive ever done for the USA. One of their efforts, in particular, McCubbin and Delucchi [38] estimated the annualized societal cost of motor-vehicle use for the year, 1990–1991 (report #11). The authors estimated the annualized social costs of motor-vehicle use (e.g. that attributed to fuel, vehicle maintenance, highway maintenance, salaries of police officers, travel time, noise, injuries from accidents), and that associated with the disease from four criteria pollutants (carbon monoxide, nitrogen dioxide, ozone, and particulate matter) and six "toxic" air pollutants (formaldehyde, acetaldehyde, benzene, 1,3-butadiene, gasoline particulates, and diesel particulates). The study considered a variety of health effects (mortality, different kind of morbidities, work days loss, restricted activity days etc.) for the whole USA. The authors further classified and estimated costs attributed to six general categories: personal non-monetary costs, motor vehicle goods and services priced in the private sector, motor-vehicle goods and services bundled in the private sector, motor-vehicle goods and service provided by government, monetary externalities, and non-monetary externalities. Personal non-monetary costs were defined by those un-priced costs of motor-vehicle use that a person imposed on him or herself as a result of the decision to travel.

The authors used exposure-response functions to estimate health damage costs and found highest costs attributed to health related costs. They dig down further and estimated the number and type of health effects and the monetized the value of these effects, including total dollar costs per kg of pollutant emitted. For most pollutants and health effects, the authors calculated upper and lower bound estimates of the effects of exposure.

To calculate the monetary valuation of health damage costs of air pollution, McCubbin and Delucchi [38] followed contingent valuation approach. Whereas the cost of the criteria pollutants was estimated on the basis of human epidemiological studies and ambient air-quality data, the cost of toxic air pollutants was estimated on the basis of unit-risk values and exposure to pollution in micro-environments. Unit-risk functions were related the probability of getting a particular type of cancer (e.g., leukemia) to the amount of exposure to a particular toxic air pollutant (e.g., benzene). To calculate the economic valuation of health effects, this report not only considered the different kinds of morbidity and symptoms of illness but also estimated lost of work days, restricted activities and mortality, and so on. While the authors estimate the value of life (VOL), which is the most important valuation parameter in the analysis, the authors distinguished future deaths from current deaths, and deaths that would have occurred soon anyway even if there were no pollution from deaths that would not have. The authors assumed that air pollution mainly kills the elderly, and that the VOL of the elderly is a less than the VOL of the middle aged working males for whom VOLs typically are derived. However, some commentators recognized that that the VOL for the elderly might be an order of magnitude lower than the VOL for young people [39,40]. One of the important finding of this report was that the largest cost of air pollution related with particulate matter and the potentially large contribution of motor vehicles to ambient particulate levels. Most of the results of the study were based on a 10% reduction in motor vehicles emission. However, for the points of reference, the study also estimated the health effects associated with either a 100% reduction in motor vehicle emissions or a 100% reduction in all anthropogenic emissions. Notice that, this study focused on a 10% reduction because it seems more useful for policy makers to consider the effect of a relatively small reduction in emissions.

The authors concluded that by far the largest environmental externality costs attributed to the particulate air pollution and the estimated total societal costs in each of the six cost categories, and the range were $885 billion (the high bound) and $267 billon (for the lower bound). The study also reported that the health cost of motor-vehicle related air pollution was about $450 billion (upper bound). However, due to suspicions in their estimates they were rather uncertain with results, even were upper bound. The author reported that most of the damages from particulates were related with mortality and chronic illness. Even though there is considerable uncertainty in the estimator process, it was clear that damages from particulates were dominated the total cost of the health effects of motor-vehicle air pollution (based o a wide range of assumptions) and the highest share of this cost was associated with mortality, which was so costly. They also estimated the effects of a specific, "marginal" change in pollution: the difference between actual pollution and, what pollution would have been had there been either with a 10% or a 100% reduction in motor vehicle-related emissions.

In another study conducted at the Institute of Transportation Studies, University of California, Delucchi *et al*. [41] estimated impair visibility cost of air pollution based on the USA data for 1990. The study estimated the cost of both the health and visibility effects of air pollution. To estimate a relationship between housing prices and housing attributes, including air quality, the authors developed a meta-hedonic price analysis (meta-HPA), based on the study of Smith and Huang [42]. Though the authors recognized that it was difficult, on the basis of HPA alone, to disentangle the health and visibility components of air quality, however, based on some reasonable assumptions about the allocation of these costs, they compared these HPA estimates of the health and visibility costs with estimates derived from alternative models. For the health effects, the authors compared their meta-HPA estimates with recent damage-function (DF) estimated by McCubbin and Delucchi [43] for the visibility effects, they evaluated their meta-HPA estimates with the results of a simple contingent valuation model (CVM) proposed by Chestnut and Dennis [44]. They summarized the total social cost of motor-vehicle pollution between $24 and $450 billion attributed to the health hazards, between $5 and $37 billion to the visibility costs, between $4 and $8 billion to material-damage costs, and between $2 and $6 billion to damages associated with forests and crops per year. The authors therefore recognized that the visibility costs were about an order of magnitude smaller than health costs, but large enough to be worth estimating.

The study also found that direct PM and SO_x _emissions had the largest visibility costs. Another important finding of this study was that meta-hedonic price analysis produces an estimate of the health cost that lies at the low end of the range of the damage-function estimates. This observation is consistent with the hypothesis that on the one hand, hedonic price analysis does not capture all of the health costs of air pollution (because individuals may not be fully informed about all of the health effects), and that on the other hand, the value of mortality used in the high-end damage function estimates is too high [41].

### Studies based on non-OECD countries

The nine non-OECD studies [1,2,45-51] also used a prevalence-based framework. Most of them investigated a single pollutant (see Table 2). However, as can be seen in Table 2, in contrast to the OECD studies already described, most of the non-OECD studies considered both the morbidity and the mortality impacts of air pollution [e.g. [2] and [46-50]]. The DRF approach was used to quantify health impacts in most of the studies. Regarding cost components, all studies estimated direct and indirect costs, but none estimated non-direct health care costs. Only six studies (one unpublished) estimated intangible costs, using the WTP approach [[1,2,46-48], &[51]], and none reported this cost component separately.

Productivity losses due to morbidities were estimated using either the HCA or the WTP approach, and costs related to premature mortality were estimated using either the HCA [e.g. [49]] or VOSL. To estimate a monetary valuation of VOSL, all studies used the simple adjustment of transferring benefits to account for the income differential between the country of interest and the country in which the VOSL data was collected [e.g. [27,29-33]]. For example, Larson *et al*. [45] and Alberini and Krupnick [1] used per capita GDP/income differentials to transfer the US VOSL into the Russian and the Taiwanese contexts, respectively, while (with some adjustments) Quah and Boon [50] imputed the UK VOSL (€1.5 million in 1992 prices) into the Singaporean context (the methodology applied by Pearce and Crowards [52]). However, it has been argued that the differing social and welfare systems in different countries may immensely influence the risk perception of the local population, and thus result in a different WTP to avoid risk [[46] Hence, the simplistic tactic of using income differences to estimate VOSL for a particular country based on the US values may be flawed, and could produce overestimates. One of the fundamental reasons for this overestimation could be that WTP for the reduction of risk rises with income.

## Key issues in the estimation of air pollution costs

The key issues in the estimation of CAP include study design and data sources (prevalence or incidence, top-down or bottom-up), cost components and their estimation, discount rate, treatment of uncertainty, and the question of attributable and avoidable cost. These issues are broadly related to two different concerns: how to identify all physical health impacts, and how these impacts can be converted into a monetary value. This section illustrates these issues and critically discusses how CAP can be affected by employing diverse approaches.

### Identification and quantification of health impacts

All the studies reviewed in this paper were prevalence-based, and the majority either took a solely top-down approach, or combined a top-down approach with a bottom-up approach. All studies based on dose-response function (DRF) to estimate the relative risks of air pollutants. To be brief, the DRF associates the quantity of a pollutant that affects a population to the physical impact on this population and a health impact can be quantified only if the corresponding DRF is known. By definition a DRF starts at the origin, and in most cases it increases monotonically with dose. According to current knowledge, the population-level DRFs for health impacts of the conventional air pollutants (NOx, PM, and SO_2_) appear to be linear without threshold, and a single calculation is sufficient [53].

However, for many pollutants and many impacts of the DRFs are very uncertain or not even known at all. For most substances and non-cancer impacts the only available information covers thresholds, though knowing thresholds is not sufficient for quantifying impacts; but rather it only provides an answer to the question whether or not there is a risk [53]. Nevertheless, if sample size is not very large then one needs relatively high doses in order to obtain observable nonzero responses. In reality such doses are generally far in excess of typical ambient concentrations in the European Union or North America. Thus there is a serious problem of how to extrapolate from the observed data towards low dos [53].

Moreover, although the applicability of this approach is context-dependent, the same techniques have been applied in different countries and settings. The difficulties with using DRFs are related not only to context, but also to temporal stability; this factor comes into play when DRF values (e.g. relative risks) are transferred from rather old US studies to developing countries in Europe and elsewhere [[22] and [54]]. Furthermore, the utility of a single DRF for a whole country may be limited for large developing countries such as India or China, due to intra-national variations in demographic composition, weather, pollution exposures, per capita income, income discrimination, and welfare systems.

### Cost components and estimation methods

As mentioned above, costs can be divided into three broad categories: direct costs, indirect costs, and intangible costs. Direct costs include both direct health care costs and direct non-health care costs. We note that cost of caregivers' time and informal care may be an important component of non-direct health care costs. However, only one [19] of the 17 studies performed a separate estimation of direct non-health care costs (work days lost due to mothers' caring for sick children); this study reported a large amount of cost associated with this component (about $833 million).

One of the more difficult issues is the estimation of productivity losses due to illness. There are two main fundamental methods for estimating productivity losses at market price due to morbidity and mortality: the HCA and the WTP approach. Both of these methods have been used in studies estimating CAP. The HCA is the most common way to estimate productivity losses. In this approach, productivity losses associated with mortality are estimated by calculating the capitalized value of future lifetime earnings that would have been earned by those who died prematurely. Average annual wages are often used to estimate the annual productivity of an average healthy person of working age. Annual productivity losses are adjusted downward to obtain "net annual productivity" (annual productivity minus the amount consumed by the worker). Productivity losses associated with morbidity are estimated by imputing the wage rates as to the value of working days lost. All the HCA based studies included in our review used wage rate data to estimate productivity losses as a measure of indirect costs. However, the HCA is associated with difficulties which in turn affect the CAP. *Firstly*, labor market imperfections may lead to unemployment, thus violating one of the assumptions of the HCA. In the context of developing countries, where the labor market is less developed, wages may not be a good measure for estimating productivity losses. *Secondly*, wage rates may not reflect the marginal productivity of workers but rather other factors, for example discrimination. *Thirdly*, CAP analysis is performed from a societal perspective, but wage rate data reflect at best the productivity of the working population, rather than the whole population, and so adjustments would also be required to correct for the proportion of the population that does not earn a wage [8]. This implies that a person's earnings may differ from the actual value of his/her output or productivity. It has also been argued that the HCA underestimates true productivity loss, because it values life using the market price, and so yields low values for people outside the labor force, such as children or retirees [55].

The WTP approach is usually used to value non-market attributes and attempts to elicit this value through the use of household surveys [56]. In this approach, hypothetical scenarios are offered to respondents, and values are assigned to health changes based on what the individual is willing to pay to prevent or avoid a disease in order to remain at their original utility level but in a healthy state [57]. Theoretically, the WTP approach has the advantage of acquiring the full range of personal cost associated with the illness. However, it has been criticized in the context of "existence" values, which do not derive from private consumption of a good [58]. It has also been noted that the results are sometimes subject to personal interpretations of the questions, and can be biased by the respondents' desire to engage in strategic behavior [59]. WTP results may also differ due to differences in the design of different questionnaires on the same illnesses [e.g. [20] and [22]].

Intangible costs reflect the patient's level of pain and suffering, and the limitations that this pain and suffering imposes on quality of life. Most of the studies included in this review used WTP to capture this cost component, and so were not able to separate it from production losses. However, although the relative contribution from different cost components of CAP studies could be useful for policy rationale, it should be remembered that double counting may arise if the individual considers all cost components in assessing their WTP for avoiding illness and a separate cost component is also imputed, for example, for production losses using wage rate data or for losses associated with quality of life [8]. It should also be mentioned that the best studies have been aware of this problem and have not in fact double counted anything significant [e.g., [53] and [60]].

Since costs due to pain and suffering cannot be judged by the HCA, studies which follow this approach simply ignore this cost component. Although costs related to reduced quality of life are difficult to estimate, there are several tools or instruments that may be used separately (e.g. the EQ-5D, the SF36, etc). In particular, the EQ-5D questionnaires give a utility value between 0.0 (dead) and 1.0 (perfect health) based on five attributes: mobility, self-care, usual activity, pain/discomfort, and anxiety/depression [8]. The number of quality-adjusted life years (QALYs) lost due to a specific disease can be calculated by comparing the difference in utility between a sample with the disease and the general population for different age groups. A monetary value can be imputed for each QALY lost in order to estimate the intangible costs [11]. An alternative measure of intangible costs could be Disability Adjusted Life-Years (DALYs), which however is a controversial concept, at least in its early form [e.g., [61,62]].

If costs are incurred at different time points, future monetary values of costs must be calculated at present values and a social discount rate would be required. The rationale for discounting or at to which rate(s) would be used in the environmental context have been described in detail in European commission [63] and Friedrich and Bickel [64]. To be brief, there are two ways in which a social discount rate can be estimated [53]. Firstly, by calculating the social rate of time preference, attempts to measure the rate which social welfare or utility of consumption declines over time. Secondly, by estimating the social marginal opportunity cost of capital which can be derived by subtracting external costs of the productive capital and adding the external benefits [53]. In the existence of efficient markets with no taxes or subsidies, the two measures would be equated by the market interest rate. In Particular, in the EU the value for the social opportunity cost of capital found to average around 6%. Combining estimates for the social time preference rates with social opportunity cost, ExternE [53] recommended three discount rates such as low (0%), central (3%) and high (6%).

Our review found that while some studies did report the discount rate, different studies used different discount rates (e.g. Vergana et al [25] used 3%, Larson et al [45] used 10%, and Srivastava & Kumar [2] use 5%), although a discount rate of 5% is often used. The use of such a typical discount rate may be helpful for comparisons over studies, but may not truly reflect individual or societal time preferences. Assuming different probable discount rates and conducting a sensitivity analysis may improve the quality of studies and allow researchers to better appraise the results [8].

Finally, the negative impacts of illness have a number of external negative effects on the affected households and society. For example, the additional health care cost may require households to alter their patterns of consumption (e.g. diet, housing, etc), saving, investment, and labor allocation [9]. Furthermore, ill-health may also increase depression and other psychological problems in the household, and also distress society in different ways. Even if these costs are difficult to measure, they should be kept in mind when estimating CAP. However, these issues are not even discussed in any of the studies included in our review.

### Treatment of uncertainty

Owing to a general paucity of information, one of the most complex issues is to estimate the uncertainty of environmental impacts and damage costs. To account for this concern, studies usually use Monte Carlo analysis of the input parameters of the damage cost calculation attributed to air pollution damages. It is appropriate for many applications, in particular air pollution damages, the Monte Carlo method is powerful; capable of treating any problem but it is difficult to see how the result would change if the input parameters are changed and it is purely numerical Spadaro and Rabl [65].

As an alternative to Monte Carlo approach, a simple and transparent alternative- estimating the uncertainties of the input parameters of the damage cost calculation for air pollutants, recently, Spadaro and Rabl [65] estimated the total uncertainty for the impacts and damage costs. The key finding was that the uncertainty of pollution damage cost can be characterized, to good approximation, by lognormal distributions (for further technical discussion, see Spadaro and Rabl [65]). The authors also determined the confidence intervals of the result for mortality, and have been found to have the largest cost in the recent estimates of air pollution [e.g. [33,34,53]].

The uncertainty of dose-response functions varies widely from study to study. With the dose-response functions, many studies encounter several kinds of problems. The confidence intervals of DRFs for health impacts are usually reported for the 95% probability, and they are approximately symmetric around the mean. However, it is acknowledged that the underlying probability distribution are usually not lognormal, therefore it is required to estimate the analogous geometric standard deviations and most of the studies did not have functions for every plausible health effect [65]. Another form of uncertainty arises when one pollutant comes from primary and others form secondary sources then it would be difficult to damage calculation. To deal with this issue, Spadaro and Rabl [65] introduced a factor for the respective toxicities of primary particles, nitrates and sulfates relative to ambient PM, and they assumed geometric standard deviations for these toxicities.

Moreover, most of the physical hazards can usually be valued by their price on market (e.g. the price of drugs or hospital days). Though there is modest uncertainty of these prices at any particular place and time, however, uncertainty comes mainly from their future assessment, particularly related on the choice of discount rates.

### Counterfactual scenarios, attributable and avoidable cost

In CAP studies, it may be important to make a comparison between the actual exposure level and some hypothetical level (e.g. a fixed or zero level of exposure to air pollution), known as the counterfactual scenario [66]. It is also useful to estimate the costs associated with some marginal change in emissions, e.g., a 10% reduction as conducted by the Institute of Transportation Studies by using the USA data [e.g. [60]]. Exposure to a health risk factor (e.g. air pollution) and a risk distribution (e.g. the effect of the disease on the individual and population, and the costs thus incurred) will lead to various economic effects for both the individual and society, both at the present time and in the future. The economic impact or costs of modified exposure to risk factors and subsequent changes in population health may be estimated using the setting of counterfactual scenarios [67]. A recent WHO document describes four counterfactual situations: the *theoretical minimum*, the *plausible minimum*, the *feasible minimum*, and the *cost-effective minimum*. The first one is defined as the exposure that would result in the lowest population risk while the second is the lowest imaginable exposure. Feasible exposure denotes the lowest exposure that has been observed in comparable populations and the cost-effective exposure is that would result if all existing cost-effective interventions are applied [68,69]. The distributional transitions, the definition of the attributable fraction, the unavoidable costs (costs resulting from previous exposure), and the avoidable fraction, based on different counterfactual situations, can be presented graphically [see [69]].

As discussed above, in the estimation of the effect of air pollution on health, it is important to decide which counterfactual situation of exposure should be considered as the threshold level. In existing research, the standard assumption is the theoretical minimum, but there are factors that may limit the use of such an approach. Firstly, in reality, a reduction to the lowest risk scenario, in which a risk condition is completely removed, will likely to be impossible to achieve, and may also be redundant from an economic point of view (due to e.g. cost-effectiveness) [70]. Secondly, the time frame needed for the distributional transition may be very long. In other words, there may be a large difference between the attributable costs at the start of the study and the avoidable costs during a set period [67,68,71]. An estimation of avoidable costs requires a combination of the attributable costs and estimates of effectiveness of interventions as well as assessments of the general development of the costs [69].

## Discussion and conclusion

The present paper conducts a systematic review focusing on the total societal costs associated with air pollution-related ill health (CAP). Following the selection criteria, we reviewed 17 CAP studies from 14 countries (see tables 1, 2, 3). Of the selected studies, eight considered mortality and morbidity effects of air pollution, eight considered only morbidity, and one study considered only mortality effects. A majority of the studies focused on the effect of a single air pollutant, PM_10_. A number of studies based on the ExternE methodology and studies based on the USA data produced by the Institute of Transportation Studies, University of California (UoC), Davis were also summarized separately.

Most of the studies found air pollutants to be the main sources of respiratory and cardiovascular diseases, and also contributory factors for hypersensitivity (different allergies, headache, eye irritation, cough, phlegm, etc.). We also found that most of the published and unpublished studies estimated the direct and indirect costs associated with air pollution, they did not consider all cost components within these categories. The majority of the studies estimated the cost of productivity losses due to both mortality and morbidity. Only two studies performed separate estimations of the intangible costs; these studies reported a considerable cost associated with pain and suffering (about $5 billion for Canada and US $46.84 on average per capita for France). Thus, it is difficult to draw conclusions about the relative burden of intangible costs associated with air pollution.

Our review reveals that in estimating CAP, different authors assume different values for the parameters used in the estimation (e.g. different relative risks for different countries). Moreover, it is important to realize that the result of a CAP estimation depends on a country's overall health care system, particularly on the institutional structure, such as the rules for sickness absenteeism, the health care financing system, and so on. For example, for salaried workers in France, wage losses are compensated by social security after thirty days of absence from work due to illness [18].

Different studies also used different methods and cost components. Though the ExternE methodology ("Impact Pathway") seems to be more complete and structured, it is not consistent for comparisons over time (even for a single country), as the different studies used different values for VOSL at different time points. Though unnoticed in the most of the studies, however, some the USA and Europe based studies [e.g. [53] &[60]] also estimated air pollution related costs attributed to crop and forest damages, visibility impairment, building damages (the studies reported a huge amount of cost associated with these components) and included with the total cost. Therefore, meaningful comparison of the monetary estimates of the reviewed studies is difficult. Keeping in mind these limitations, we observe that air pollution-related health hazards have a huge societal cost. For example, CAP was approximately 3.4% of GDP in Singapore and 1% of GDP in Jakarta. Based on ExternE estimates, pollution-related damage cost was about 2.8% of GDP for Germany, 4.4% for Italy, 3.9% for the Netherlands, and 2.0% for the UK. Table 3 presents calculations in per capita terms (in US dollars) for the CAP estimations of the different studies included in the current review. The per capita cost ranged between $6 and $613 in the OECD countries and $0.20 and $2000 in the non-OECD countries. The figures seem to be higher for non-OECD than OECD countries because, for example, in estimating CAP Saksena & Dayal [49] consider both outdoor and indoor air pollution (but do not report the cost of indoor pollution separately).

Mortality seemed to be the dominant fraction of the CAP. How should mortality be monetized? Two main approaches are discussed in the current literature; value of a statistical life (VOSL), and value of a life year (VOLY). Though most recent ExternE studies considered VOLY, however, all studies in our review used VOSL as the basis for the estimation of premature mortality cost. It should be noted that VOSL does not seem appropriate here, as most VOSLs are derived from young or middle-aged people, while most air pollution victims are children and elderly people. Therefore, VOLY could produce more correct estimates of mortality cost because it considers the age structure difference between the air pollution-sensitive population and the total population, and also follows the welfare theoretic basis of WTP [37]. Another argument for VOLY is that VOSL is mostly relevant for accidental death, and is not appropriate for mortality caused by air pollution, since air pollution cannot be determined or identified as the cause of any individual death as it is not a primary cause of death but rather a contributory one [72]. A significant step towards dealing with this issue was taken by Krupnick *et al*. [73], who developed a specific questionnaire for contingent valuation studies of air pollution mortality, and then applied this questionnaire in countries such as Canada, Japan, and the USA. The ExternE project has also used the same questionnaire very recently in France, Italy, and the UK; based on the results from these countries, ExternE projects currently use a VOLY of €50,000 for Europe [53].

Moreover, ambient PM, including sulfates, nitrates, and organic aerosols, accounts for about 95% of the total damage cost, and mortality related to ambient PM accounts for about 70% of the total damage cost. Consequently, assumptions about the relationship between PM and mortality, and about the value of mortality, strongly determine the overall cost estimates. The considerable uncertainty in these two relationships leads to great uncertainties in the total cost estimates as well [38]. For example, the differences between the estimates of McCubbin and Delucchi [38,43], and those made by Krupnick and Portney [74], Hall *et al*. [75], and Small and Kazimi [76], can be explained largely by different assumptions regarding the number of deaths attributable to PM pollution, and the value of those deaths. For the value of a statistical life, in particular, Hall *et al*. [75] assumed a range of $1.8 to $9.2 million, Small and Kazimi [76] assumed a range of $2.0 to $11.0 million, and Krupnick and Portney [74] assumed a value of $1.0 million (on the presumption that air pollution kills old and sick person with a low value of life). In a survey of 26 studies (21 labor market studies, 5 contingent valuation studies), the USEPA found that the average value of a statistical life was $4.8 million in 1990 dollars (USEPA, 1999). McCubbin and Delucchi [38,43] use a range of $0.01 to $0.05 million for air-pollution related deaths that would have occurred very soon anyway, had there been no pollution, and $1.0 to $4.0 million for deaths that would not have occurred soon [38]. In these studies, there were also large differences in the estimates of the number of deaths related to PM pollution. In the recent ExternE project, an analysis of the external costs of air pollution from power plants and motor vehicles in Europe has been discussed and the problem of valuing a statistical life was divided in two parts: the number of life-years lost due to air pollution, multiplied by the value of a life-year lost [77]. However, in the ExternE analysis, the number of life years lost either was assumed or else was estimated on the basis of the Pope *et al*. [78] cohort epidemiology study, and the value of a life-year was estimated on the basis of a mid-range assumption regarding the value of a statistical life (about $3 million). Though uncertainty in ExternE was split into different components, however, the uncertainty was not eliminated or even substantially reduced yet.

With few exceptions [e.g. [1,18,19,21] etc.] the majority of the studies reviewed here considered the health effects of a single air pollutant, PM_10_. However, it is well-documented that human health is adversely affected by five criteria pollutants, namely sulfur oxide (SO_2_), carbon monoxide (CO), nitrogen dioxide (NO_2_), particulate matter (especially PM_10 _and PM_2.5_), and ozone. Thus, many environmental specialists believe that the true number of air pollution-related health problems has been underestimated. Moreover, most of the studies used the annual average level of air pollutant in their analysis, while in reality pollution levels fluctuate quite widely on an hourly basis. Some studies have also shown that the air pollution level at peak hours (i.e. from 8 am to 5 pm) is much higher than the annual average level. Consequently, the estimated health impact of air pollution or societal cost would depend on which pollution level is used.

Most of the studies focus only human health damages due to air pollution and ignores injure crops and forest, damage building materials, visibility degradation and ecosystem. Analysis based in the USA has conducted that reduced visibility is one of the major impacts of air pollution [53]. In Europe, however, the issue has received little attention. New research has been undertaken in the USA, most notably by the Institute of Transportation Studies, UoC, Davis and ABT Associates [79]. In particular, Delucchi and colleagues [41] had used meta-hedonic price approach (meta-HPA) to estimate heath and other damage costs. They reported $45–37 billion per year attributed to the visibility costs, $0.4–8.0 billion to the material-damage costs, and $2–6 billion to the forests and crops damage costs. They also noticed that though the visibility costs were found smaller than the health costs, however, large enough absolutely to be worth estimating. Moreover, given the lack of concern about air pollution hazards related with the visibility damage in Europe [80], one option would be to consider these costs by using related values based on the USA data. However, to evaluate policies regarding visibility effects of air pollution in Europe that the values based on the USA experience would be inappropriate due to the uncertainties involved in the benefit transfer. In the absence of a specific contingent valuation study for Europe aiming to elicit the average WTP measure to improve visibility, some adjustment in the USA numbers may be done to account for this lower concern about visibility effects. Though it is not clear on what basis this might be done, recently ExternE [53] also reported crop damage costs by air pollution about 3,874 million Euro.

All studies included in our review focused solely on outdoor pollution; however, in developed countries, 89% of all person-hours are spent in indoor environments, leaving only 11% of time to be spent outdoors, while in developing countries the corresponding proportions are 70% and 30% [50]. Moreover, in developing countries, the most part of a woman's time is spent indoors. Women in developing countries spend most of their time caring for babies, collecting fuel, and cooking; they also normally use solid fuels (such as wood, dung cake, coal etc.) for cooking under poor ventilation conditions [49]. Cooking and heating with solid fuels on open fire or traditional stoves results in high levels of indoor pollution. Indoor smoke also contains a range of health damaging pollutants (PM, CO, SO_2_, and so on) and two major health hazards are associated with these pollutants, namely acute lower respiratory infections in young children aged under five, and chronic obstructive pulmonary disease in adults over twenty [81]. Consequently, many mothers and children (age < 5 years) in rural areas suffer from respiratory symptoms or illness. Although indoor air pollution affects health adversely, no single study in our review provided separate cost estimates for outdoor and indoor air pollution.

Only one study [21] considered the effect of outdoor air pollution on asthma for children aged between 1 and 18 years. The important finding of this paper was that the net effect of pollution is larger for children of lower socio-economic status. It should be noted that neurobiological and economic research has suggested that early shocks to a child's health can persist for many years [82,83]. Therefore, if poorer families are unable to afford to live in cleaner areas, their children's health development will suffer. This would indicate that environmental pollution may be one potential mechanism by which socio-economic status affects health.

For policy purposes, the distinction between 'avoidable' and 'unavoidable' cost estimates may be more important than reporting the total or full cost of air pollution-related health hazards. However, although there are some important reasons for estimating 'avoidable' costs separately, no study has attempted to split the total cost into 'avoidable' and 'unavoidable' parts. Separating 'avoidable' costs from the full cost may help governments and policy makers to decide what preventions or policies will be most effective at reducing the economic impact of air pollution-related health hazards.

## Conclusion

It is evident that none of the current studies is complete, as they all omit some measurable cost components and each has some limitations with respect to the methods used in estimating CAP. The results of the reviewed studies show considerable variation, due to differences in exposure level and differences in the health care systems of different countries. Moreover, due to differences in the data and methods, CAP estimates are not comparable across studies. Nevertheless, some important insights emerge from the analysis of the results obtained from our review. This review highlights the large uncertainties involved in estimating CAP. In particular, there are uncertainties about the existence of health effects and there are statistical uncertainties about the values of coefficients. Due to variations in the methodologies and uncertainties in the estimated costs, the current CAP studies seem to be limited for policy analyses. To increase awareness concerning the air pollution-related burden of disease, and to build a link to health policy analyses, future research efforts should be directed towards theoretically sound and comprehensive CAP studies with use of rich data. The final section of this paper contains some specific suggestions.

### Future research

As expected, we have identified a number of gaps and limitations in existing research. Based on these, we recommend that future research should include all key cost components; moreover, rather than using lower bound estimates, which represent a "conservative" figure, all best estimates should be reported, and quantifications should be made with theoretical justification. A more explicit approach should be followed to deal with uncertainties in the estimations, for example using the Bayesian methods that allow explicitly for the integration of subjective assessments, multivariate sensitivity analyses (i.e. simulate the estimates by imputing the plausible ranges of values of different parameters) should be employed [14]. To reduce uncertainties in physical health impacts, the appropriate approach should be followed, specifically, by setting up a DRF for single pollutant (e.g. PM_10_) and health effects. Monetary valuations should also be imputed by the country's own estimates, and different socio-economic groups within the country must be taken into consideration (e.g. to estimate productivity losses). For example, different wages for different income groups as well as different pollution exposures for different socio-economic groups within the society should be considered [2]. If these factors are not taken into account, there is a risk of either underestimating or overestimating the number of health problems, and consequently the societal cost associated with air pollution.

To understand the consequences of air pollution and to estimate accurate societal cost, future research should also be directed towards producing better data, preferably longitudinal data. To calculate the precise incidence rate (mortality or morbidity) due to specific air pollution, it would be necessary to estimate population attributable fraction (PAF) for every country, in line with the WHO recommendation that relative risks should be estimated based on data from the specific country itself, rather than relying on other epidemiological studies. To date, air pollution-related COI studies have mostly used a prevalence-based COI framework. To fill this important gap and to make CAP studies useful for policy analysis, future research efforts should, if possible, use an incidence-based CAP framework [14].

In addition, authors should not only present their results on the monetary values of health hazards, but should also illustrate all physical impacts that are caused by air pollution, for example, number of restricted activity days (RADs), number of life years lost (LYL), and so on. This will facilitate comparisons of health impacts over time and across countries, for researchers as well as policy makers, even if the unit values of health hazards differ between different time points within countries or across countries. Moreover, VOLY, rather than VOSL, should be used to evaluate mortality cost, following the recent estimates based on the questionnaire designed specifically by Krupnick *et al*. [73] for the contingent valuation of air pollution mortality. In particular, this approach makes it possible to handle the overestimation problem in the premature mortality costs of air pollution, and this will help to produce the most credible estimates of societal costs.

Future CAP studies should not only provide aggregate cost estimates, but should also report source-specific cost estimates, following the example of the recent ExternE studies which found that most of the total costs were caused by petrol and diesel cars. Though typically in estimating source-specific costs are very expensive and time consuming and it is particularly difficult in developing countries settings, where resources are scarce. However, such source-specific cost estimates would be great help in efficient resource allocation, providing an important basis for priority-setting within this risk factor and cost-effectiveness analyses of alternative policy interventions for the key causes.

While the setting-up of a threshold level (or counterfactual situation) is an important issue, there is currently no scientific basis for setting a particular threshold in evaluations of the health impact of air pollution [46]. However, the existing literature does contain a number of different suggestions for where such a threshold should be set, including a zero threshold, the natural background level, the lowest observed level in epidemiological studies, and standards established by law or policy, such as the standards set by the USEPA, or by the WHO, or other country-level standards. According to the WHO, for example, even if no man-made pollution existed, every country in the world would have at least 10 μg particles concentration for per cubic meter; and so this level can be considered as the 'background concentration' [84]. For future CAP studies, the best alternative would be to use different threshold assumptions and perform sensitivity analyses. Finally, for policy reasons it is important to know the 'avoidable cost' attributable to CAP, and so future research should attempt to develop an appropriate method for estimating such costs.

## Competing interests

The authors declare that they have no competing interests.

## Authors' contributions

TP and UGG conceived the study. TP, UGG, and CHL designed the study. TP analyzed and wrote the paper. All authors' provided significant comments, wrote, read, and approved the final manuscript.
